# Genetics of Pediatric Epilepsy: Next-Generation Sequencing in Clinical Practice

**DOI:** 10.3390/genes13081466

**Published:** 2022-08-17

**Authors:** Antonela Blazekovic, Kristina Gotovac Jercic, Sarah Meglaj, Vlasta Duranovic, Igor Prpic, Bernarda Lozic, Masa Malenica, Silvana Markovic, Lucija Lujic, Zeljka Petelin Gadze, Romana Gjergja Juraski, Nina Barišic, Ivo Baric, Fran Borovecki

**Affiliations:** 1 Department for Functional Genomics, Center for Translational and Clinical Research, University Hospital Center Zagreb, University of Zagreb School of Medicine, 10000 Zagreb, Croatia; 2 Department for Anatomy and Clinical Anatomy, University of Zagreb School of Medicine, 10000 Zagreb, Croatia; 3 Department of Neurology, University Hospital Center Zagreb, University of Zagreb School of Medicine, 10000 Zagreb, Croatia; 4 Department of Neuropediatrics, Children’s Hospital Zagreb, 10000 Zagreb, Croatia; 5 Department of Pediatrics, Clinical Hospital Center Rijeka, Faculty of Medicine, University of Rijeka, 51000 Rijeka, Croatia; 6 Department of Pediatrics, University Hospital of Split, University of Split School of Medicine, 21000 Split, Croatia; 7 Department of Pediatrics, University Hospital Center Sestre Milosrdnice, 10000 Zagreb, Croatia; 8 Dr. Tomislav Bardek General Hospital Koprivnica, 48000 Koprivnica, Croatia; 9 Referral Centre of the Ministry of Health of the Republic of Croatia for Epilepsy, Affiliated to ERN EpiCARE, 10000 Zagreb, Croatia; 10 Neuropediatric Clinic, Srebrnjak Children’s Hospital, 10000 Zagreb, Croatia; 11 Department of Pediatrics, University Hospital Center Zagreb, 10000 Zagreb, Croatia

**Keywords:** epilepsy, pediatric, next-generation sequencing, gene panel, clinical decision making

## Abstract

Epilepsy is one of the most common neurological disorders with diverse phenotypic characteristics and high genetic heterogeneity. Epilepsy often occurs in childhood, so timely diagnosis and adequate therapy are crucial for preserving quality of life and unhindered development of a child. Next-generation-sequencing (NGS)-based tools have shown potential in increasing diagnostic yield. The primary objective of this study was to evaluate the impact of genetic testing and to investigate the diagnostic utility of targeted gene panel sequencing. This retrospective cohort study included 277 patients aged 6 months to 17 years undergoing NGS with an epilepsy panel covering 142 genes. Of 118 variants detected, 38 (32.2%) were not described in the literature. We identified 64 pathogenic or likely pathogenic variants with an overall diagnostic yield of 23.1%. We showed a significantly higher diagnostic yield in patients with developmental delay (28.9%). Furthermore, we showed that patients with variants reported as pathogenic presented with seizures at a younger age, which led to the conclusion that such children should be included in genomic diagnostic procedures as soon as possible to achieve a correct diagnosis in a timely manner, potentially leading to better treatment and avoidance of unnecessary procedures. Describing and discovering the genetic background of the disease not only leads to a better understanding of the mechanisms of the disorder but also opens the possibility of more precise and individualized treatment based on stratified medicine.

## 1. Introduction

Epilepsy is a heterogeneous disease with numerous clinical manifestations and various causes underlying the disease [1]. Part of the fundamental disease pathways is known, but many epilepsy cases remain of unclear etiology. With the development of genomic approaches, there has been a shift towards a more precise and accurate diagnosis of epilepsy by primarily researching the genetic background of symptom development [2]. Clinicians are trying to shift away from the “one size fits all” approach to the treatment of epilepsies. It has been shown that treatment based on symptoms and semiology of seizures is not always successful and that the percentage of refractory epilepsies is still high [3]. This was also recognized by the ILAE, and a shift can be seen in the classification of diseases, leading to the disease etiology being divided into six categories, one of which is genetic [4]. This underlines the importance of an etiology-based management approach.

For decades, it has been perceived that at least some people with epilepsy have a genetic background, and this was first confirmed in families with epileptic syndromes [5]. Today, it is estimated that 70–80% of epilepsy cases have genetic variants underlying the disease that are at least partly responsible for the onset of symptoms. Moreover, 20–30% can be considered secondary forms due to acquired conditions such as stroke, brain trauma, and tumors [6]. As with other complex diseases, a smaller proportion can be explained by monogenic changes while the remaining cases are caused by a complex interplay of environmental and multiple genetic factors. The application of genomic tests in children with epilepsy has led to the identification of new causative genes and expanded knowledge about the biological basis of epilepsy, which may also lead to therapeutic implications [1].

This study aimed to investigate the genetic etiology of pediatric epilepsy cases and to show the importance of targeted genomic tests in the diagnostic workflow of epilepsy.

## 2. Materials and Methods

### 2.1. Participants

We retrospectively collected data from 277 cases of epilepsy patients aged 6 months to 17 years referred to the Department for Functional Genomics, Center for Translational and Clinical Research, University Hospital Center Zagreb, and University of Zagreb, School of Medicine, from 2016 to 2020. Exclusion criteria included seizures caused by acquired brain injury (including traumatic brain injury and neoplasm).

All patients were examined and diagnosed by a neurologist and/or pediatrician. We analyzed clinical data, including age at the onset of the disease, frequency, and type of seizures, as well as the presence of developmental delay and cranial malformations, if present. Seizure types and epileptic syndromes have been diagnosed and classified according to the guidelines of the International League Against Epilepsy (2014, 2017) [4]. We also assessed whether the patients had refractory epilepsy and whether epilepsy was present as part of a syndrome. All procedures were in accordance with the 1964 Helsinki Declaration and its later amendments, or comparable ethical standards.

### 2.2. Sample and Data Collection

Blood samples (3 mL) were collected in EDTA-containing tubes from all patients and control subjects. DNA was extracted from whole blood samples following the manufacturer’s specifications using the Quick DNA Kit (Zymo Research, Irvine, CA, USA). A NanoDrop 2000 (Thermo Fisher Scientific, Waltham, MA, USA) spectrophotometer and a Qubit 4 (Invitrogen) fluorometer were used to determine the quality and concentration of genomic DNA samples. Only DNA of OD (optical density coefficient, 260/280) 1.80 ± 20% was used for further experiments.

### 2.3. Next-Generation Sequencing

The data for this study was obtained by next-generation sequencing of the custom epilepsy gene panel consisting of 142 genes (listed in Supplementary Table S1). The genes were selected through a comprehensive search of the literature (PubMed), and clinical databases of human genes and genetic disorders (OMIM, Clin-Var, and HGMD), and the genes associated with epilepsy were included. The panel included the coding DNA sequences, except for the *CSTB* gene, where the 5′UTR region was included, and for the *TSC1*, *TSC2*, *NF1*, and *NF2* genes, where all introns were also covered, libraries for next-generation sequencing were prepared using Nextera XT DNA Library Prep Kit (Illumina, San Diego, CA, USA) according to manufacturer’s instructions. Briefly, each DNA sample was diluted, fragmented, and amplified. The amplified samples were re-purified with magnetic particles, and the quality and quantification of the samples were evaluated via a DNA 1000 Kit using Agilent 2100 Bioanalyzer (Agilent, Santa Clara, CA, USA). Next, the fragments were hybridized and amplified, and the regions of interest were separated using streptavidin magnetic particles. The next step was to add double indexes to name each sample for further recognition, and finally, they were diluted to a concentration of 12 pM.

The libraries prepared were sequenced on a MiSeq (Illumina) and NextSeq 550 (Illumina) next-generation sequencing platform according to the manufacturer’s instructions, generating approximately 5 million of the 150 bp paired-end reads for each sample (Q30 ≥ 96%, average passing filter 89.30%) with a mean region coverage depth of minimum 80.0 (median 125.5) and a median target coverage at 20× of 93%, and at 50× of 83.4%. After sequencing, the FASTQ files were imported to Variant Interpreter (Illumina), which was used for further analysis of the sequencing data. After sequencing data submission, the pipeline included the following steps: quality checks and filter of the reads; alignment on the reference genome, coverage statistics, and metrics; and variant calling and annotation. We focused on two variant types; single nucleotide polymorphisms (SNPs) and short insertions and deletions (indels). Alignment mapping and variant calling in our analysis pipelines were performed with the Variant Interpreter (Illumina) and DRAGEN (Illumina) platforms. Annotation was followed by the filtering and classification of variants depending on phenotype and clinical indication for testing. All variants were classified according to the guidelines jointly established by the American College of Medical Genetics and Genomics (ACMG) and the Association for Molecular Pathology for the interpretation of sequence variants [7]. The final interpretation of the variant was ultimately guided by the phenotype of the case. Variants were reported based on relevance to the primary indication for testing. All likely pathogenic and pathogenic variants are listed in the supplement data (Supplementary Table S2).

### 2.4. Statistical Analysis

Differences between different patient characteristics and the rate of reported variants were compared through Chi-squared or ANOVA tests depending on the data. All statistical analyses were performed with the Statistica 10 software (StatSoft, Tulsa, AK, USA), and a *p*-value of <0.05 was considered statistically significant.

## 3. Results

Our cohort consisted of 277 participants with unexplained pediatric-onset epilepsy referred to our Department for Functional Genomics from 2016 to 2020. The majority of cases were without identifiable genetic syndrome (n = 231; 83%). For the remainder of the cohort, almost half of the participants presented with craniofacial malformations and/or developmental delay (n = 115; 49.8%). Age at enrollment was 4 weeks to 17 years, with seizure onset at birth to 16 years of age (median = 2 years, 30% < 1 year). Fifty-two percent of participants were male (144/277). All participants were Caucasians of Croatian origin. The main demographic data are presented in Table 1.

**Figure 1:**
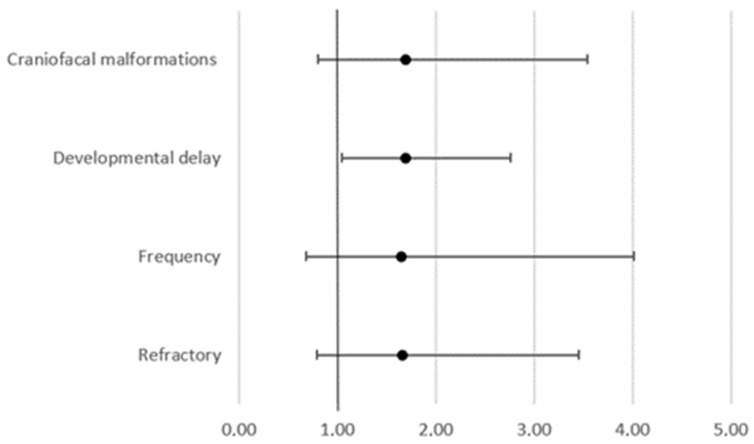
Odds ratio for patient’s characteristics between patients with a negative NGS panel result and patients with variants reported as VUS, LP, or P.

We detected 118 variants in 38.6% of our study participants (107/277). Of those 118 variants, 54 were described as variants of unknown significance (VUS), 47 were described as likely pathogenic (LP), and 17 were described as pathogenic (P). The overall yield of pathogenic and likely pathogenic variants from our cohort was 23.1% (n = 64). The highest diagnostic rate achieved in our study was among participants with epilepsy accompanied by developmental delay with pathogenic or likely pathogenic variants in 28.9% (n = 42/146) (Table 1). Further analysis showed that developmental delay can be related to the presence or absence of a mutation (chi-square, 4.538, *p* = 0.03315) but not how the mutation is reported (chi-square, 3.43, *p* = 0.179962.). On the other hand, there was no correlation between craniofacial malformations, and the presence or absence of a mutation (chi-square, 1.9736, *p* = 0.16007). Furthermore, there was a significant difference in the odds of having a developmental delay between patients with a negative NGS panel result and patients with variants. This was reported as VUS, LP, or P (OD 1.6963; 95% CI 1.0414 to 2.7629; *p* = 0.0337), but there is no significant difference in the odds for craniofacial malformations (95% CI 0.8299 to 3.0415; *p* = 0.1623) or for refractory epilepsy (95% CI 0.9579 to 1.2999; *p* = 0.1593) (Figure 1).

The overall rate of reported variants did not differ significantly between participants divided according to the frequency of symptoms (chi-square, 1.984, *p* = 0.370842) or the presence of refractory epilepsy (chi-square, 1.8533, *p* = 0.173402). There was also no significant difference in the overall rate of reported variants depending on epilepsy type (chi-square, 5.1893, *p* = 0.158447).

**Figure 2:**
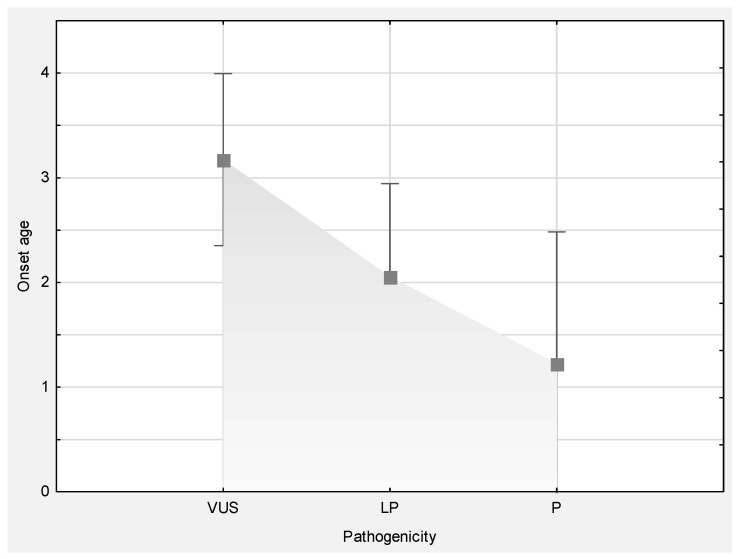
Significantly different age at onset between groups of patients stratified according to variant annotation.

As previously mentioned, the age at enrollment was 4 weeks to 17 years, with seizure onset between birth and 16 years of age (median = 2 years, 30% < 1 year). Interestingly, pathogenicity report significantly correlated with age at onset (one-way ANOVA F (2.77) = 3.7887, *p* = 0.02695), meaning that patients with variants reported as pathogenic had a younger age of onset than patients with variants described as VUS (Figure 2).

Although variants were analyzed with a panel consisting of 142 genes, variants in only 47 genes have been detected in this group of 277 patients. Of 118 variants described, 38 (32.2%) were not described in the literature. Those 38 variants were present in 25 genes. One novel variant in the *ALDH7A1* gene, 1566-1G>T, rs140845195) was present in four patients. The genes with repeated novel variants were *ADGRV1*, *ALDH7A1*, *KCNT1*, *MBD5*, *SCN2A*, *SCN8A*, *TBC1D24*, and *TSC2*.

**Figure 3:**
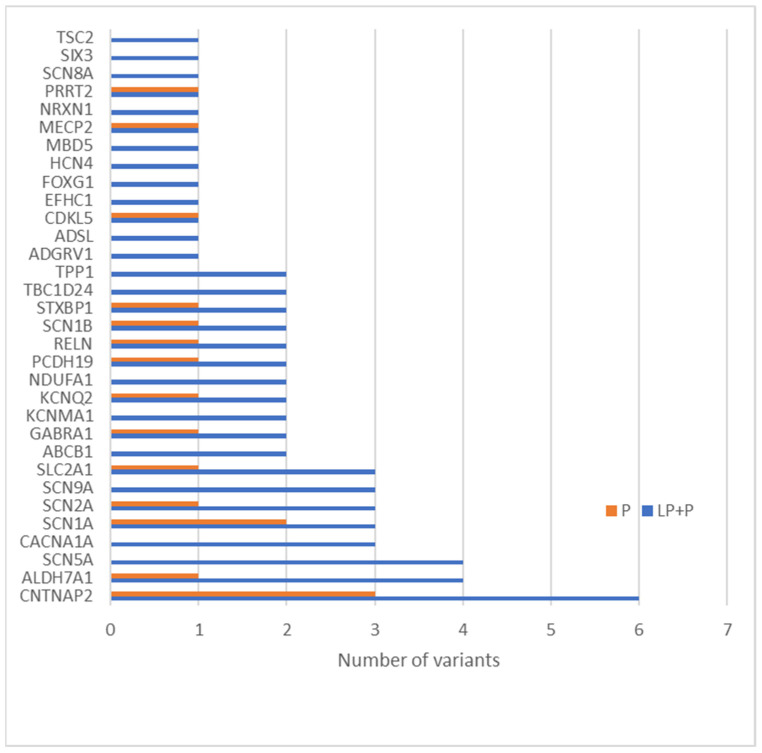
Sixty-four candidate variants classified as LP or P in 32 genes in our group.

**Figure 4:**
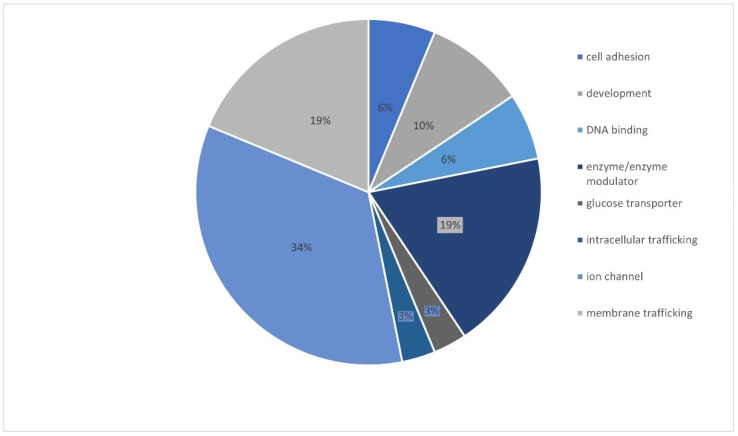
Functional classification of the mutated pathogenic or likely pathogenic genes.

The most clinically interesting group of variants were the ones described as pathogenic and likely pathogenic. There were altogether 47 variants described as LP and 17 as P. These 64 variants were related to 32 genes, and the gene with the most frequent variants was *CNTNAP2* (Figure 3). Most genetic changes (34.38%) have been identified in genes encoding ion channels (*CACNA1A*, *GABRA1*, *SCN1B*, *SCN5A*, *SCN1A, HCN4, KCNQ2, SCN9A, SCN2A*, and *SCN8A*), but variants have also been reported in genes encoding enzymes and/or enzyme modulators (18.75%) (*TSC2, NDUFA1, ALDH7A1*, *TPP1*, *ADSL*, and *CDKL5*) as well as in genes associated with membrane trafficking(18.75%) (*CNTNAP2*, *ABCB1*, *ADGRV1*, *STXBP1*, *EFHC1*, and *PRRT2*) (Figure 4).

## 4. Discussion

In the recent decade, the development and greater availability of advanced genomic methods have led to a major shift towards etiological diagnostics of epilepsy. The number of causal genes is increasing daily, which has led to an increase in diagnostic yield [8]. The overall yield of pathogenic or likely pathogenic variants from our cohort was 23.1%. Miao and colleagues had a slightly higher yield of 27% but included only pathogenic variants [9]. It is important to note that they had comparable inclusion and exclusion criteria but used a larger panel of 480 genes compared to ours consisting of 142 genes [9]. On the other hand, Rochtus and colleagues achieved a higher yield and identified pathogenic or likely pathogenic variants in 40% (50/125) of their study participants [10]. The difference in yield can be explained by the use of WES in this study, but perhaps an even more important difference is that the profile of patients was slightly different and that the cohort consisted of as many as 70% of patients with developmental delay and epileptic encephalopathy [10]. On the other hand, Hoelz and colleagues showed a diagnostic yield of 18% reporting LP and P variants [11]. The importance of the number and selection of genes included in the panel was shown by Parrini and colleagues. They showed that a 95-gene panel allowed for a genetic diagnosis in 6.3% of patients that would have otherwise been missed using a 30-gene panel [12]. Ortega-Moreno found disease-causing variants in 19.5% of analyzed patients using two panels of 83 and 106 genes [13]. Another study that showed the importance of a good selection of suitable patients but also the importance of a purposeful diagnostic course was conducted by Patel et al. [14]. They showed that patients with genetic causes of epileptic encephalopathy were diagnosed by clinical features, metabolic investigations, MRI, or microarray in 44% of cases, targeted next-generation sequencing epilepsy panels in another 44% of cases, and whole exome sequencing in 12% of cases [14]. Our data together with the results of other studies show that the diagnostic yield depends on the adequate selection of patients and the number of genes analyzed. An increase in the number of genes analyzed is not proportionally linked to an increase in the diagnostic yield; thus, for the majority of patients, it would not be cost-effective to perform WES. Parrini and colleagues concluded that panels with about 100 genes represent the best cost-effective diagnostic method in drug-resistant pediatric epilepsy [12].

It is crucial to understand the great importance of including advanced genomic methods in the diagnostic process of pediatric epilepsy. Identifying genetic causes can help select appropriate pharmacotherapeutic approaches, may lead to fewer (often invasive) diagnostic methods required, and may lead to timely use of other non-AED therapies. Hoelz et al. showed that 63% of patients with reported LP or P variants experienced changes in clinical management and avoided further diagnostic evaluation after genomic testing [11]. Our study showed a significantly higher diagnostic yield in patients with developmental delay as well as in patients with disease onset at a very young age, which leads to the conclusion that such children should be included in genomic diagnostics as soon as possible to achieve the correct diagnosis in a timely manner, to potentially improve treatment, and to prevent unnecessary diagnostic procedures.

Miao and colleagues found the largest number of variants in the *SCN1A* gene, followed by *KCNQ2*, *KCNT1*, and *PCDH19* [9]. Parrini and colleagues found that *SCN2A* was the most frequently mutated gene, followed by *SCN1A*, *KCNQ2*, *STXBP1*, *SCN8A*, *CDKL5*, and *MECP2* [12]. Variants found by Ortega-Moreno were all in known epilepsy-associated genes (*KCNQ2*, *CDKL5*, *STXBP1*, *SCN1A*, *PCDH19*, *POLG*, *SLC2A1*, *ARX*, *ALG13*, *CHD2*, *SYNGAP1*, and *GRIN1*) [13]. All listed genes were among the most prevalent genes in our cohort, but the gene with the largest number of variants in our cohort was *CNTNAP2*, which was not present in the large epi panel of Miao and colleagues [9], or in other smaller aforementioned panels [10,11,12,13]. This shows that not only the size of the panel but also the careful selection of the genes included in the panel that are important. Around a third of genetic changes (34.38%) have been identified in genes encoding ion channels (*CACNA1A*, *GABRA1*, *SCN1B*, *SCN5A*, *SCN1A*, *HCN4*, *KCNQ2*, *SCN9A*, *SCN2A*, and *SCN8A*), but variants have also been reported in genes encoding enzymes and/or enzyme modulators (*TSC2*, *NDUFA1*, *ALDH7A1*, *TPP1*, *ADSL*, and *CDKL5*) as well as in genes associated with membrane trafficking (*CNTNAP2*, *ABCB1*, *ADGRV1*, *STXBP1*, *EFHC1*, and *PRRT2*) and cell divisions and processes (*SIX3*, *FOXG1*, and *RELN*), which is in accordance with other studies [2]. Furthermore, this study confirmed that mutations in one gene can lead to a spectrum of epilepsy phenotypes and that the same variants can exhibit different phenotypes. For instance, variant 1566-1G>T in the ALDH7A1 gene is a novel variant and was present in four patients with different symptoms. Interestingly we found some more novel variants present in more patients. Variant c.1361_1362delAT in *CNTNAP2* was also present in four patients. The *CNTNAP2* gene encodes for a neuronal transmembrane protein member of the neurexin superfamily, Contactin-associated protein-like 2 (CASPR2), which clusters voltage-gated potassium channels at the nodes of Ranvier and is involved in neuron–glia interactions [15]. Mutations in the *CNTNAP2* disrupt the expression of CASPR2, causing abnormalities in cortical histogenesis. Such mutations have been identified in neurodevelopmental disorders such as autism, intellectual disability, and epilepsy [16]. In a study from 2006, Strauss et al. showed that homozygous mutation in *CNTNAP2* causes cortical dysplasia-focal epilepsy (CDFE), a disorder that results in epileptic seizures, intellectual disability; hyperactivity; speech regression; and in most cases, autism [17].

Pathogenic variants were identified in several genes, with *SCN1A, SCN2A,* and *KCNQ2*, being the most significant ones. The *SCN1A* and *SCN2A* mutations result in truncated sodium channels, which alter the transmission of depolarizing impulses throughout the neurons. *SCN1A* mutations are associated with developmental and epileptic encephalopathy, Dravet syndrome, febrile seizures, and generalized epilepsy with febrile seizures plus (GEFS+) [18,19,20]. *SCN2A* mutations are associated with developmental and epileptic encephalopathy [21,22], episodic ataxia [23], and benign familial infantile seizures [24]. The mechanism of pathogenicity of these mutations remains unknown, but it is suggested that gain-of-function and loss-of-function defects contribute to abnormal neuronal network excitability [21]. On the other hand, *KCNQ2* encodes voltage-gated potassium channel subunit, which together with the KCNQ3 subunit can form neuronal M channels that carry slowly activating and non-inactivating potassium currents. These currents contribute to resting membrane potential and regulate excitability in central and peripheral neurons. *KCNQ2* mutations are associated with developmental and epileptic encephalopathy, benign neonatal seizures, and myokymia [25]. Loss-of-function mutations in either subunit cause neuronal hyperexcitability and can lead to benign familial neonatal convulsions. Even though the underlying pathogenetic mechanism of epileptic encephalopathy remains unclear, it is believed that a combination of defects in voltage-dependent activation and axonal expression causes a decrease in potassium current and its ability to inhibit neuronal excitability in the brain [26]. The limitations of our study are that we did not perform genotype–phenotype correlations and parental DNA analysis. By performing complex and precise genotype–phenotype correlations after the NGS epilepsy gene panel, Horák and colleagues increased their diagnostic yield by 53.33% [27]. Furthermore, we did not perform re-testing of samples. Salinas and colleagues detected pathogenic variants initially in 38% of subjects with developmental and epileptic encephalopathies. However, after an average time of 29 months, 25% of the subjects without a genetic diagnosis were re-categorized and diagnosed [8]. Finally, patients were not monitored throughout the study, and we cannot access data from the diagnostic test to monitor the impact on therapeutic management.

## 5. Conclusions

This research has shown the importance of including advanced genomic methods in the diagnostic process of pediatric epilepsy. This study has presented that at least a quarter of pediatric epilepsy patients previously classified as patients with unknown etiology has a clear genetic etiology. We have shown that a good selection of patients who would benefit the most from genetic diagnostics is extremely important because patients who developed epilepsy at a younger age and who have a developmental delay in addition to epilepsy are most likely to have a causal genetic variant detected by advanced genomic methods. We have also shown the importance of the proper selection of genes to be included in the panel. However, from a diagnostic point of view, it should be noted that not every genomic method is good for every patient and the choice of the most appropriate genetic test can play a pivotal role. In conclusion, an ideal diagnostic pipeline would consist of a good clinical examination by a pediatrician and a clinical geneticist who, if necessary, refer the patient to a well-designed epilepsy panel. If a pathogenic or likely pathogenic variant is detected, genotypic–phenotypic tests should also be performed, and it is recommended to include them in the diagnostic workflow. Furthermore, in an ideal situation, an analysis of the parents (the so-called case-parent triad) should also be carried out.

## Figures and Tables

**Table 1:** Descriptive statistics.

	**Total Cohort (n = 277)**	**VUS ^1^ (n = 54)**	**LP ^1^ (n = 47)**	**P ^1^ (n = 17)**
Male/female (%)	52/48	61.1/38.9	42.6/57.4	47.1/52.9
Mean age at sample sequencing (range)	8.4 (0.5–17.5)	8.8 (1–17.5)	7.7 (0.5–17)	7 (1–14)
Mean age at disease onset (range)	3 (0–15.5)	3.2 (0–12)	2.1 (0–10.5)	1.2 (0–4)
Focal epilepsy	14.1%	9.3%	12.8%	11.8%
Generalized epilepsy	41.2%	40.7%	57.4%	47.1%
Combined epilepsy	34.3%	37.0%	23.4%	35.3%
Syndrome	16.6%	13.0%	19.1%	29.4%
Refractory	51.6%	55.6%	57.4%	17.6%
Speech delay	13.7%	9.3%	27.7%	52.9%
Learning disability	10.1%	11.1%	17.0%	52.9%
Behavioral changes	6.9%	13.0%	4.3%	5.9%
Psychomotor retardation	27.4%	25.9%	38.3%	0.0%
Craniofacial malformations	11.6%	18.5%	14.9%	0.0%
Abnormal EEG	72.6%	83.3%	55.3%	41.2%

^1^ VUS, variants of unknown significance; LP, likely pathogenic; P, pathogenic.

## Data Availability

The data presented in this study are available from the corresponding author upon request.

## Supplementary Materials

The following supporting information can be downloaded at: https://www.mdpi.com/article/10.3390/genes13081466/s1, Table S1: List of genes included in the custom epilepsy gene panel; Table S2: Variants classified as likely pathogenic or pathogenic.
