# Supplementary material for: Genetics of Pediatric Epilepsy: Next-Generation Sequencing in Clinical Practice
Source: Genes (Basel). 2022 Aug 17;13(8):1466. doi: 10.3390/genes13081466 (PMC9407986; doi:10.3390/genes13081466)
Supplement: Supplementary file 1 [file genes-13-01466-s001.zip › genes-1800327-supplementary.pdf]

Suppl. Table S1. List of genes included in the custom epilepsy gene panel

ABAT	ABCB1	ADSL	ALDH7A1	ARFGEF2	ARHGEF9
ARX	ASPM	ATN1	ATP1A2	ATP6AP2	ATR
BCKDK	CACNA1A	CACNB4	CASK	CASR	CCL2
CDK5RAP2	CDKL5	CDON	CENPJ	CEP152	CHRNA2
CHRNA4	CHRNA7	CHRNB2	CLCN2	CLN3	CLN5
CLN6	CLN8	CNTNAP2	CPA6	CSTB	CTSA
CTSD	DCX	DNAJC5	EFHC1	EMX2	EPM2A
FKTN	FLNA	FLVCR2	FOLR1	FOXG1	FOXH1
GABRA1	GABRB3	GABRD	GABRG2	GAMT	GATM
GLI2	GOSR2	GPR56	GPR98	GRIN1	GRIN2A
GRIN2B	HCN1	HCN4	KANSL1	KCNA1	KCNAB1
KCNMA1	KCNQ2	KCNQ3	KCNT1	KCNJ10	KCNJ11
KCTD7	LGI1	LIAS	MAGI2	MAPK10	MBD5
MCPH1	MECP2	MEF2C	MFSD8	MTHFR	NDE1
NDUFA1	NF1	NF2	NHLRC1	NODAL	NOTCH3
NRXN1	OPHN1	PAFAH1B1	PCDH19	PCNT	PHF6
PLCB1	PNKP	PNPO	POLG	PPT1	PRICKLE1
PRICKLE2	PRRT2	PTCH1	RELN	SCARB2	SCN1A
SCN1B	SCN2A	SCN3A	SCN5A	SCN8A	SCN9A
SHH	SIX3	SLC19A3	SLC25A19	SLC25A22	SLC2A1
SLC9A6	SPTAN1	SRPX2	ST3GAL3	ST3GAL5	STIL
STXBP1	SYN1	TBC1D24	TCF4	TGIF1	TPP1
TSC1	TSC2	TSEN2	TSEN34	UBE3A	VANGL1
WDR62	XRCC1	ZEB2	ZIC2.		

The panel includes the coding DNA sequences, except for the CSTB gene, where the 5`UTR region is included, and for the TSC1, TSC2, NF1 and NF2 genes, where also all introns are covered

Suppl. Table 2. Variants classified as likely pathogenic or pathogenic

Gene	Variant-c.	Variant-p.	Chromosome	rs	HOM/HET	Change	Pathogenicity
NRXN1	569A>G	Asn190Ser	2:51254843	200792504	het	missensse	LP
PCDH19	1549G>A	Ala517Thr	X:99662047	778072039	het	missensse	LP
SIX3	29A>C	Tyr10Ser	2:45169272		het	missensse	LP
FOXG1	587delA	Gln196ArgfsTer17	14:29237072		het	frameshift	LP
RELN	2015C>T	Pro672Leu	7:103281044	201044262	het	missensse	LP
MBD5	236G>A	Gly79Glu	2:149221327	34995577	het	missensse	LP
MECP2	799C>T	Arg267Ter	X:153296516	61749721	het	missensse	P
TSC2	2242G>A	Glu748Lys	16:2122871		het	missensse	LP
NDUFA1	94G>C	Gly32Arg	X:119005968	1801316	het	missensse	LP
ALDH7A1	1566-1G>T		5:125880712	140845195	het	splice	LP
TPP1	622C>T	Arg208Ter	11:6638271	119455955	het	stop/gain	LP
ADSL	340T>C	Tyr114His	22:40746022	37425953	het	missensse	LP
CDKL5	533G>A	Arg178Gln	X:18602452	267606715	het	missensse	P
SLC2A1	470G>A	Gly157Asp	1:43396343		het	missensse	LP
TBC1D24	1008delT	His336GlnfsTer12	16:2548263	398122967	het	frameshift	LP
CACNA1A	2708delG	Arg903ProfsTer170	19:13409751		het	frameshift	LP
GABRA1	640C>T	Arg214Cys	5:161309644	727503940	het	missensse	LP
SCN1B	374G>A	Arg125His	19:35524569	759839781	het	missensse	LP
KCNMA1	34A>G	Ser12Gly	10:79397367	77602559	het	missensse	LP
SCN5A	4057G>A	Val1353Met	3:38601826	199473233	het	missensse	LP
SCN1A	1043G>A	Gly348Glu	2:166904264		het	missensse	LP
HCN4	2275G>A	Val759Ile	15:73616159	62641689	het	missensse	LP
KCNQ2	2171delC	Pro724ArgfsTer206	20:62038445		het	frameshift	LP
SCN9A	1964A>G	Lys655Arg	2:167138296	121908919	het	missensse	LP
SCN2A	4825A>G	Met1609Val	2:166245141		het	missensse	LP
SCN8A	1475G>A	Arg492His	12:52100339	750170064	het	missensse	LP
CNTNAP2	1361_1362delAT	Asn454ArgfsTer24	7:146997245		hom	frameshift	LP
ABCB1	1105A>G	Ile369Val	7:87180049	199766539	het	missensse	LP
ADGRV1	6556C>G	Pro2186Ala	5:89985743		het	nonsense	LP
STXBP1	1000A>T	Met334Leu	9:130434366		het	missensse	LP
EFHC1	545G>A	Arg182His	6:52303361	3804505	het	missensse	LP
PRRT2	649dupC	Arg217ProfsTer8	16:29825015	587778771	het	frameshift	P
